# Assessing the community-level impact of group antenatal care on uptake of intermittent preventive treatment for malaria in pregnancy in Atlantique Department, Benin, 2021–2023: a cluster randomized controlled trial

**DOI:** 10.1186/s12936-025-05412-8

**Published:** 2025-07-01

**Authors:** Julie R. Gutman, Faustin Onikpo, Manzidatou Alao, Julie Niemczura, Stephanie Suhowatsky, Julie Buekens, Marie Adeyemi, Katherine Wolf, Catherine Dentinger, Alexandre Binazon, Audrey S. Eunice Amoussou, Odette Aimée Alihounou, Courtney Emerson, Ahmed Saadani Hassani, Houetohossou Camille, Cyriaque D. Affoukou, Peter J. Winch, Aurore Ogouyèmi-Hounto

**Affiliations:** 1 Malaria Branch, Division of Parasitic Diseases and Malaria, Centers for Disease Control and Prevention, Malaria Branch, Global Health Center, 1600 Clifton Rd. NE, Atlanta, GA 30322 USA; 2 U.S. President’s Malaria Initiative Impact Malaria Project, MCD Global Health, Cotonou, Benin; 3 U.S. President’s Malaria Initiative Impact Malaria Project, MCD Global Health, Silver Spring, MD USA; 4 U.S. President’s Malaria Initiative Impact Malaria Project, Jhpiego, Baltimore, MD USA; 5 Malaria Branch, U.S. President’s Malaria Initiative, CDC, Atlanta, GA USA; 6 U.S. President’s Malaria Initiative, CDC, Cotonou, Benin; 7 Ministry of Health, Cotonou, Benin; 8 U.S. President’s Malaria Initiative Impact Malaria Project/Department of International Health, Johns Hopkins Bloomberg School of Public Health, Baltimore, MD USA; 9 Unité de Parasitologie/Faculté des Sciences de la Santé, Université d’Abomey Calavi, Cotonou, Benin

**Keywords:** Malaria, Pregnancy, Prevention, Antenatal care, Benin, Group ANC

## Abstract

**Background:**

In 2023, an estimated 36 million pregnancies occurred in malaria endemic sub-Saharan Africa, but only 44% received the WHO recommended ≥ 3 doses of intermittent preventive treatment (IPTp3). Group Antenatal Care (G-ANC) is a service delivery model associated with higher quality of and greater retention in ANC, in which pregnant women are enrolled into groups at their first ANC visit and subsequent care is provided in groups. A cluster-randomized controlled trial was conducted in Atlantique Department, Benin, to assess whether G-ANC improved ANC retention and IPTp3 uptake at community level.

**Methods:**

Forty purposively selected health facilities (HF) were randomized 1:1 to control (individual ANC) or G-ANC. Cross-sectional household surveys to measure uptake of ANC and IPTp were conducted in each HF catchment area before and after implementation among randomly selected women who had given birth in the previous 12 months. Changes in coverage were assessed using a difference-in-difference approach, adjusting for HF clustering.

**Results:**

At baseline (N = 1259), coverage of at least 4 ANC visits (ANC4) and IPTp3 was 52.8% and 48.0%, respectively, in the intervention catchment, and 44.9% and 49.4% in the control catchment. Coverage of ANC4 improved in both arms by endline (N = 1280), to 56.7% in the intervention and 46.1% in the control, but the difference in the increase was not significant between arms (p = 0.51). Coverage of IPTp3 increased non-significantly (p = 0.26), to 53.2% (intervention) and 49.7% (control). Overall, only 140 (10.6%) surveyed women reported participating in G-ANC. Participation improved coverage of both ANC4 (65.0% vs 50.5%, p = 0.002; odds ratio (OR) 1.9, 95% CI 1.4–2.5) and IPTp3 (64.0 vs 50.6%, p = 0.004; OR = 1.8, 95% CI 1.2–2.6).

**Conclusions:**

G-ANC increased ANC attendance and IPTp3 uptake among women who participated, but participation was limited. Understanding and addressing the barriers to participation is critical if G-ANC is to be used more widely to increase IPTp coverage.

*Trial Registration*: PACTR202405487752509

**Supplementary Information:**

The online version contains supplementary material available at 10.1186/s12936-025-05412-8.

## Background

Malaria remains an important cause of morbidity and mortality in sub-Saharan Africa (SSA). In 2023, 36 million pregnant women in the World Health Organization (WHO) African Region were estimated to be at risk for malaria, with 12.4 million affected during pregnancy [1]. To combat the adverse effects of malaria in pregnancy, the WHO recommends that HIV-negative pregnant women in malaria endemic areas receive at least three doses of intermittent preventive treatment in pregnancy (IPTp) with sulfadoxine-pyrimethamine (SP) starting as early as possible in the second trimester, with doses given at least 1 month apart [2]. Despite recent improvements, IPTp uptake remains inadequate; in 2023 only 44% of pregnant women in sub-Saharan Africa received the recommended three doses [1]. A number of factors contribute to the low uptake including: sub-optimal antenatal care (ANC) utilization (in 2022 only 55.5% of pregnant women in SSA made four or more ANC visits) [3], SP stock-outs, provider workload and confusion regarding efficacy of IPTp-SP, and client misconceptions [4, 5].

Group Antenatal Care (G-ANC) is a promising health system intervention recommended by WHO as a potential alternative to individual ANC in the context of rigorous research [6]. In this model, women with pregnancies of similar gestational age are offered enrolment in a group during the first ANC visit, and return for subsequent care in scheduled group meetings, facilitated by an ANC service provider. Group sessions are highly participatory and encourage women to learn, share, and problem-solve together. G-ANC has been found to be more effective than individual ANC at retaining women in care and increasing utilization of health services (including a higher number of ANC visits, more facility-based births, higher coverage of ANC-delivered interventions, including IPTp), longer gestation, and higher birthweight [7–9]. G-ANC is associated with improved health literacy and high levels of satisfaction among both women and providers [10–12].

Early initiation of ANC is critical for the success of G-ANC. Benin was selected for this study as nearly half of pregnant women presented for first ANC (ANC1) prior to the fourth month, with 83% of all pregnant women attending at least 1 visit and 51% attending at least 4 visits [13]. Despite this, IPTp coverage is low with only 13.7% of women receiving at least three doses of IPTp (IPTp3 +) [13]. This study assessed whether implementation of the G-ANC model improved retention in and quality of ANC, and whether it was feasible to scale the intervention sufficiently to have an impact measurable at the community level- the target was to enroll at least 50% of ANC1 clients into G-ANC in intervention facilities. Specifically, the study aimed to determine if the implementation of group ANC led to a higher proportion of recently pregnant women, surveyed at the community level, who received the recommended three or more doses of IPTp (IPTp3 +) as compared to communities where the facilities provided only individual ANC, the conventional service delivery model in Benin.

## Methods

A longitudinal cluster-randomized controlled trial was conducted in the three health zones (HZ) of Atlantique Department, Benin, from 2020 to 2022 to assess whether coverage of IPTp increased in catchment areas of health facilities (HF) conducting G-ANC compared with those providing conventional, individual ANC (i.e., the current standard of care). The trial was registered at PACTR, number 202405487752509.

### Study setting

Atlantique Department, located in south-central Benin, is the most populous of the 12 administrative departments of Benin, with a population of 1.3 million people. The predominant ethnic group is Fon, and Christianity is the most common religion. In Atlantique in 2021–2022, 97.8% of women attended ANC, 65.9% of whom initiated prior to 4 months gestational age. Two-thirds (66.5%) attended at least four visits. However, IPTp3 coverage was only 21.7% [14].

Study health facilities were selected from among 89 facilities providing ANC in three of eight HZs of Atlantique Department. Initially 51 facilities were deemed suitable (i.e., a public or not-for-profit maternity department with an average minimum of 20 and maximum of 130 ANC1 visits per month, easily accessible at all times of year). Site eligibility was reassessed after delays due to the COVID-19 pandemic, and only 40 facilities met criteria (including adequate space to hold a meeting considering COVID-19 limitations). All were included in the study. Intervention and control facilities were assigned 1:1 using restricted randomization accounting for IPTp3 + coverage measured by the baseline survey. (Fig. S1, Map of Atlantique Dept with intervention and control sites). Blinding was not possible due to the nature of the intervention.

### G-ANC implementation

Enrolment: In intervention facilities, pregnant women attending their first ANC visit prior to 25 weeks gestational age and intending to remain in the study area for the duration of the study were offered the opportunity to enroll and receive subsequent ANC care in scheduled monthly meetings. Pregnant women presenting for their first ANC visit after 25 weeks of gestation and those not consenting to G-ANC were excluded, and received standard, individual ANC. Each G-ANC cohort included 8–15 women of similar gestational age, scheduled for five 90-min, highly participatory meetings, at 4-week intervals (Fig. S2). The ANC provider conducted a private clinical consultation and physical exam with each participant at every G-ANC meeting, in addition to group activities. SP was administered by directly observed therapy to each participant during the private clinical consultation. While individual pregnant women were consented and enrolled into GANC, the study did not collect individual-level data on women from the ANC registers in either intervention or control facilities. Rather, assessment was based on data collected during baseline and endline household surveys, described below. See supplemental methods for more details.

Training, monitoring, and supervision: ANC providers at all 40 study sites participated in a 1-day ANC technical update between December 2020 and March 2021 to standardize basic care, including IPTp and gestational age estimation. In addition, 52 intervention facility providers received a 5-day highly participatory didactic and practical training on G-ANC facilitation in February 2021. Two health officials per HZ also participated in the training. Study staff conducted monthly and quarterly visits to intervention and control facilities, respectively, to monitor progress and support providers. See supplemental methods for more details.

### Baseline and endline cross-sectional surveys

Implementation of cross-sectional surveys: Experienced staff were hired and trained for 4 days to conduct baseline and endline household surveys during November 2-December 12, 2020, and October 24-December 2, 2022, respectively. Health facility catchment areas were mapped through conversations with health facility and ministry staff. One randomly selected enumeration area (EA, according to the National Statistical Institute (INSAE) geographic delineations) per health facility was mapped. During mapping, staff documented which households included a woman aged 15–49 who had experienced a live birth in the previous 12 months (recently pregnant women), and 33 of these households were randomly selected per EA. If an EA had too few households, two adjacent EAs were combined. Very large EAs were segmented, and one segment was mapped. After obtaining written consent from the recently pregnant woman, study staff administered a standardized questionnaire in French or the local language Fon [Fon-gbe] about demographics, malaria prevention, and ANC experiences offered, and collected responses on a tablet in CommCare (V2.52, Dimagi, Inc.). Households in which residents were absent were revisited at least twice before being declared absent. Absent households were not replaced.

Sample size: The sample size for the cross-sectional surveys was calculated to assess the community-level impact of implementing G-ANC on IPTp3 uptake between baseline and endline surveys. Based on data available in 2019, prior to implementation, IPTp3 coverage was estimated to be 14% nationwide [15], and it was assumed that, in absence of the intervention, IPTp3 coverage would increase to 16% over the study period. With 20 clusters per arm, assuming a 0.04 intra-cluster correlation coefficient, and allowing for a 10% survey refusal rate, a sample size of 33 women who completed a pregnancy within the prior 12 months per cluster (total 1320 women per survey) was required to detect a 10-percentage point difference between G-ANC and conventional ANC arms, with 80% power and an alpha of 5% (i.e., assuming 16% in the control arm at endline, IPTp3 coverage of 26% or greater in the intervention arm would be statistically significant).

### Survey of post-partum women

Implementation of post-partum survey: As a result of lower-than-expected participation in G-ANC, the protocol was amended in 2022 to include data collection from women at presentation in early labor or immediately postpartum at 18 intervention health facilities regarding their household malaria prevention activities and ANC. The objective of this survey was to validate the proportion of women who participated in G-ANC and the coverage of preventive interventions among women who participated. Between July-November 2022, a subset of questions from the cross-sectional household survey were administered to eligible women, i.e., women between the ages of 15–49 years who had initiated ANC prior to 25 weeks gestational age and provided written consent, irrespective of their participation in G-ANC.

Sample size: Using baseline survey results (IPTp3 + uptake of 40%), it was estimated that a sample size of 1254 women would be needed to detect a 10 percentage-point difference in IPTp3 + uptake following exposure to **G-**ANC with 80% power and alpha 0.05, assuming a design effect of 3.

### Semi-structured interviews and focus group discussions

Semi-structured interviews, lasting 60 to 90 min, were conducted with providers (i.e., midwives, nurses, and nurse’s aides) from six intervention arm health facilities, two from each of the three HZs, and HZ officials (four HZ supervisors and three chief medical officers [CMOs]) approximately 15 months after implementation began. An effort was made to conduct face-to-face interviews with all relevant personnel in each health centre, 2–3 interviews per facility. Three of the health centres saw more than 100 ANC1 clients per month and three saw fewer than 100 ANC1 clients monthly. Interviews followed a guide that explored attitudes towards providing G-ANC, perceived changes in provider communication and relationship with women, satisfaction related to the provider’s perceived ability to do their job well, perceived effects of G-ANC on fellow providers and participating women, and perceived change in uptake of IPTp. In addition, convenience sampling was used to select women who attended G-ANC more than twice during their most recent pregnancy from the same six facilities. A total of 140 women were invited to join the focus groups, and 129 participated. Focus groups, lasting 60 to 120 min, were held at intervention health facilities, in outdoor pavilions, open spaces, or meeting rooms and were conducted by two female interviewers (ASEA and OAA) trained in the social anthropology of health and qualitative research; groups included only the interviewers and participants. Interviews and focus groups were conducted in French or Fon, translated into French (as needed), and transcribed in French in a template document based on the interview guide. Transcripts were not shared with participants. Results were coded by two people (ASEA and OAA); responses were recorded in Google Forms with both numeric ratings and quotes, and findings for each module summarized following an adapted process of Framework Analysis [16, 17].

### Data management and analysis

Statistical analyses of quantitative data were performed using SAS version 9.4 (Charlotte, NC). All analyses accounted for clustering at the health facility level and were weighted to adjust for sample design. For the cross-sectional household surveys, a difference in differences analysis was conducted to estimate the change in proportion of women receiving IPTp from baseline to endline in intervention versus control facilities. Additional post-hoc analyses were conducted on both the cross sectional and post-partum survey data to assess the impact of G-ANC participation on individual outcomes. Results with a p < 0.05 were considered statistically significant. The primary outcome was the proportion of recently pregnant women at the cluster level who received 3 or more doses of IPTp (IPTp3), calculated using data from the cross-sectional surveys. Secondary outcomes, calculated at the cluster level using data from the cross-sectional surveys, included the: (1) proportion of women who received 1, 2, or 4 or more dose of IPTp; (2) proportion of women who made 1, 2, 3, 4, 6, or 8 ANC visits; (3) gestational age at the time of first ANC and at 1 st dose of IPTp; (4) proportion of women who recently gave birth who had a facility-based delivery; (5) proportion of women who recently gave birth who received quality antenatal care, defined as having blood pressure measured, a urine test, a blood test, being protected against tetanus, receiving iron and folic acid, receiving albendazole for de-worming, receiving an ITN, and receiving IPTp; and (6) mean birthweight of infants.

### Ethical considerations

The study was reviewed and approved by the *Comité National pour l'Ethique de la recherche en santé* (National Ethics Committee for Research in Health) in Benin and by the U.S. CDC Institutional Review Board. Community sensitization ensured that local leaders and community members were informed about the study. HZ officials and facility in-charges provided permission for study activities in each health centre. Signed informed consent was obtained from each person prior to their participation in G-ANC, surveys, or semi-structured interviews. Patients or the public were not involved in the design, conduct, reporting, or dissemination plans of this research.

## Results

A total of 3179 women consented to participate in G-ANC (11% of all those presenting for ANC1 at intervention facilities), and 270 groups were formed between March 2021 and December 2022. Of enrolled women, 2516 (79.1%) attended at least 1 group meeting and 1330 (52.8%) attended the fifth meeting (Table 1).

**Table 1:** G-ANC participation and retention and IPTp3 coverage by facility, 20 intervention facilities, Atlantique Department, Benin, March 2021-March 2023

Health zone	Health facility	Number of cohorts	Total enrolled	Attended ≥ 1 meeting, n(%)^	Attended 5 th meeting, n(%)^	Received IPTp3 +	Facility delivery*, n(%)	HWs trained in G-ANC^$^
AS	Kpanroun	12	140	86 (61.4%)	18 (20.9%)	34 (39.5%)	86 (61.4%)	3
	Ganvie	4	45	35 (77.8%)	0 (0%)	ND	35 (77.8%)	2
	Ouedo	31	447	435 (97.3%)	320 (73.6%)	379 (87.1%)	435 (97.3%)	4
	Zinvie	15	154	122 (79.2%)	22 (18%)	56 (45.9%)	122 (79.2%)	2
	Dekoungbe	16	167	121 (72.5%)	58 (47.9%)	77 (63.6%)	121 (72.5%)	4
	Subtotal	78	953	799 (83.8%)	418 (52.3%)	546 (68.3%)	799 (83.8%)	15
ATZ	Tangbo-Djevie	13	133	87 (65.4%)	28 (32.2%)	45 (51.7%)	87 (65.4%)	3
	Sedje-Denou	3	34	29 (85.3%)	7 (24.1%)	ND	29 (85.3%)	1
	Agon	17	180	177 (98.3%)	96 (54.2%)	121 (68.4%)	177 (98.3%)	1
	Houegbo	16	165	126 (76.4%)	85 (67.5%)	93 (73.8%)	126 (76.4%)	2
	Soyo	9	100	76 (76%)	40 (52.6%)	53 (69.7%)	76 (76%)	1
	Sehoue	18	218	163 (74.8%)	78 (47.9%)	95 (58.3%)	163 (74.8%)	1
	Dodji-Bata	16	172	84 (48.8%)	20 (23.8%)	46 (54.8%)	84 (48.8%)	2
	Togoudo	11	101	91 (90.1%)	63 (69.2%)	57 (62.6%)	91 (90.1%)	1
	Subtotal	103	1103	833 (75.5%)	417 (50.1%)	510 (61.2%)	833 (75.5%)	12
OKT	Tori-Gare	16	179	156 (87.2%)	102 (65.4%)	ND	156 (87.2%)	1
	Savi	11	144	107 (74.3%)	46 (43%)	70 (65.4%)	107 (74.3%)	1
	Avame	10	132	87 (65.9%)	52 (59.8%)	ND	87 (65.9%)	2
	Tori-Cada	15	215	180 (83.7%)	119 (66.1%)	ND	180 (83.7%)	1
	Tori-Bossito	13	173	132 (76.3%)	82 (62.1%)	103 (78%)	132 (76.3%)	3
	Kpomasse	15	169	127 (75.2%)	39 (30.7%)	67 (52.8%)	127 (75.2%)	3
	Kpovie	9	111	95 (85.6%)	55 (57.9%)	55 (57.9%)	95 (85.6%)	1
	Subtotal	89	1123	884 (78.7%)	495 (56%)	295 (33.4%)	884 (78.7%)	12
	TOTAL	270	3179	2516 (79.1%)	1330 (52.9%)	1351 (53.7%)	2516 (79.1%)	39

^Proportions are calculated out of those who attended at least one meeting; ND = not documented/illegible data

^*^G-ANC participants who delivered at the same HF, excluding those referred elsewhere; Data were abstracted from the G-ANC register

^$^These numbers do not include health aides who are not considered qualified providers

### Baseline and endline cross-sectional surveys

At the community level, 1259 women who had completed pregnancies in the past 12 months were interviewed at baseline and 1280 at endline (Table 2). Surveyed women were on average 27 years old and 97% of women were married; there were no significant differences by study arm or time point. Most women had primary education or less (78.6%, 95% CI 74.8–82.5% at baseline; 73.1%, 95% CI 69.5–76.7% at endline; p = 0.001). While there was a significant difference in educational levels from baseline to endline, there was no difference by intervention arm in either survey. The baseline survey had a significantly larger proportion of women in the lowest wealth quintile compared to the endline (15.7% (11.6–19.8%) vs 8.9% (6.4–11.5%), p < 0.0001).

**Table 2:** Demographic characteristics of women with a recent delivery (past 12 months) surveyed at baseline and endline cross-sectional surveys in catchment areas of 40 study facilities in Atlantique Department, Benin

Total	Baseline			End line		
	Control	Intervention	Total	Control	Intervention	Total
	629	630	1259	638	642	1280
Mean age, years (SD)	27.1 (6.0)	26.8 (5.7)	26.9 (5.8)	27.1 (6.1)	27.3 (6.2)	27.2 (6.2)
Married/living together, n (%)	613 (97.5)	612 (97.1)	1225 (97.3)	617 (96.7)	625 (97.4)	1242 (97.0)
Education						
No education	282 (44.8)	274 (43.5)	556 (44.2)	278 (43.6)	236 (36.8)	514 (40.2)
Primary	206 (32.8)	213 (33.8)	419 (33.3)	202 (31.7)	220 (34.3)	422 (33.0)
Secondary or higher	141 (22.4)	143 (22.7)	284 (22.6)	158 (24.8)	186 (29.0)	344 (26.9)
Slept under an ITN last night	583 (94.2)	575 (92.9)	1158 (92.0)	575 (93.7)	559 (92.7)	1134 (88.6)
Parity						
1 pregnancy	125 (19.9)	134 (21.3)	259 (20.6)	124 (19.4)	140 (21.8)	264 (20.6)
2 pregnancies	147 (23.4)	142 (22.5)	289 (23.0)	129 (20.2)	133 (20.7)	262 (20.5)
3 pregnancies	357 (56.8)	354 (56.2)	711 (56.5)	385 (60.3)	369 (57.5)	754 (58.9)
Prior pregnancies, mean (SD)	3.3 (1.9)	3.1 (1.8)	3.2 (1.9)	3.5 (2.1)	3.3 (2.0)	3.4 (2.0)
Wealth quintiles						
Lowest	212 (33.7)	187 (29.7)	399 (31.7)	148 (23.2)	97 (15.1)	245 (19.1)
2nd lowest	47 (7.5)	61 (9.7)	108 (8.6)	33 (5.2)	26 (4.1)	59 (4.6)
Middle	208 (33.1)	223 (35.4)	431 (34.2)	277 (43.4)	260 (40.5)	537 (42.0)
Second highest	100 (15.9)	102 (16.2)	202 (16.0)	130 (20.4)	168 (26.2)	298 (23.3)
Highest	62 (9.9)	57 (9.1)	119 (9.5)	50 (7.8)	91 (14.2)	141 (11.0)

Uptake of G-ANC was much lower than desired, with some contamination of the control arm; overall, only 12.9% (9.7–16.0%) of women reported having been offered participation in G-ANC and only 10.6% (8.1–13.1%) reported participating- 14.0% (10.0–18.1%) in the intervention arm and 6.5% (4.8–8.1%) in the control arm. Initiation of ANC was delayed slightly in the intervention arm at endline (difference in differences (DiD) = 0.32 months, 95% CI 0.05–0.59, p = 0.02), though this appeared to be driven more by the fact that women surveyed at baseline in the intervention arm attended ANC earlier than women in the control arm. ANC retention improved in both the control and intervention arms. The increase in total number of ANC visits was slightly, but non-significantly, greater in the intervention arm (on average 0.3 more visits, 95% CI −0.04 to 0.67, p = 0.08) (Table 3). There was a significant increase in the total number of IPTp doses among women in the intervention arm (DiD = 0.28, 95% CI 0.07–0.48, p = 0.008), and a trend toward a greater proportion of women completing IPTp3 +, though this was not statistically significant (DiD = 1.25, 95% CI 0.89–1.75, p = 0.19). There was no significant difference in timing of initiation of IPTp (Table 3), with 75% of women reporting initiation of ANC within the fourth month of pregnancy.

**Table 3:** Results include clustering by HF for baseline and endline cross-sectional surveys in catchment areas of 40 study facilities in Atlantique Department, Benin

	Recently pregnant, baseline		Recently pregnant, endline		Difference	p-value
	Control arm	Intervention arm	Control arm	Intervention arm		
ANC						
Total ANC visits	3.9 (3.7–4.2)	4.2 (4.0–4.4)	4.2 (3.9–4.4)	4.73 (4.5–4.9)	0.31 (−0.04 to 0.67)	0.08
ANC1	98.4% (97.0–99.1)	97.5% (95.6–98.6)	97.6% (94.7–99.0)	98.5% (97.0–99.2)	2.41 (0.52–11.23)	0.26
ANC2	89.0% (84.5–92.3)	91.1% (88.4–93.3)	87.3% (82.7–90.8)	91.3% (88.5–93.4)	1.20 (0.61–2.39)	0.60
ANC3	70.0% (63.4–75.9)	77.9% (72.6–82.4)	69.2% (65.7–72.5)	76.4% (72.8–79.7)	0.96 (0.58–1.57)	0.86
ANC4 +	43.8% (38.1–49.6)	52.0% (46.3–57.6)	46.0% (41.4–50.7)	57.5% (53.2–61.7)	1.14 (0.82–1.58)	0.42
ANC6 +	14.2% (11.0–18.0)	19.9% (17.2–22.9)	12.9.62–17.2)	25.2% (20.7–30.3)	1.50 (0.93–2.43)	0.09
ANC8 +	3.7% (2.4–5.8)	3.8% (2.4–6.0)	4.8% (2.8–8.1)	7.6% (5.3–10.7)	1.59 (0.70–3.62)	0.27
Participation in G-ANC	–	–	6.5% (4.8–8.1)	14.0% (10.0–18.1)	OR = 2.38 (1.58–3.56)	<.0001
Initiation of ANC (months)	3.5 (3.3.73)	3.1 (2.9–3.3)	3.4 (3.2–3.6)	3.3 (3.2–3.5)	0.32 (0.05–0.59)	0.019
IPTp						
IPTp doses	2.0 (1.9–2.2)	2.0 (1.9–2.2)	1.9 (1.7–2.0)	2.1 (2.0–2.3)	0.28 (0.07–0.48)	0.008
IPTp1	89.5% (86.9–91.7)	91.0% (88.4–93.0)	90.9% (88.4–92.8)	89.5% (87.1–91.4)	0.73 (0.49–1.08)	0.12
IPTp2	74.9% (70.7–78.6)	72.6% (67.9–76.8)	74.0% (69.5–78.0)	76.3% (72.0–80.2)	1.27 (0.88–1.84)	0.20
IPTp3	49.3% (44.0–54.7)	48.0% (44.1–51.9)	48.6% (42.5–54.8)	52.9% (46.5–59.2)	1.25 (0.89–1.75)	0.19
IPTp4	23.2% (19.1–27.8)	23.3% (19.5–27.5)	25.7% (21.6–30.3)	25.5% (20.1–31.8)	0.98 (0.65–1.50)	0.94
IPTp5 +	8.8% (6.5–12.0)	10.3% (6.8–15.2)	8.4% (6.2–11.4)	10.4% (7.2–14.8)	1.07 (0.55–2.08)	0.85
Timing of first dose of IPTp, months	4.3 (4.1–4.5)	4.1 (3.9–4.3)	4.2 (4.0–4.4)	4.1 (4.0–4.2)	0.06 (−0.21 to 0.33)	0.67

^*^Results adjusted for SES were not substantively different

A composite measure for overall quality of ANC, comprised of seven interventions (assessment of blood pressure, urine, blood, and provision of iron/folate, deworming, insecticide-treated bed nets [ITN], and at least one dose of IPTp) was relatively low, with less than 50% of women receiving all interventions (Table 4). Overall quality of care improved over time in both arms, but the improvement was statistically significant only in the control arm. There was no significant difference between arms at either time point (Table 4). A high proportion (> 95%) of women were assessed for hypertension (blood pressure measured), had a urine sample taken at least once during ANC, had a blood sample taken at least once during ANC, and received iron and folic acid, with no significant differences by time point or study arm. There were no differences in uptake of tetanus toxoid by study arm; the majority of women received at least one dose of tetanus toxoid during pregnancy, yet only about 65% overall were considered sufficiently vaccinated [18]. Receipt of deworming medication was slightly lower, at 82.7% overall, with a statistically significant difference between control (81.2%, 95% CI 78–84.3) and intervention (87.0%, 95% CI 82.8–91.2) at endline (p = 0.03). The proportion of women who received an ITN at ANC was only 61.8% on average. There was no difference in utilization of an ITN on the night prior to the survey either by arm or by time point.

**Table 4:** Quality of care received by study arm and timepoint from baseline and endline cross-sectional surveys in catchment areas of 40 study facilities in Atlantique Department, Benin

Total	Recently pregnant, baseline			Recently pregnant, endlinE		
	Control	Intervention	p-value	Control	Intervention	p-value
	629	630		638	642	
Any ANC, %	98.4 (97.0–99.1)	97.5 (95.6–98.6)		97.6 (94.7–99.0)	98.5 (97.0–99.2)	
ANC in the Public Sector only, %	88.7	85.2	0.45	88.8	80.1	0.05
ANC in the Private Sector only, %	10.3 (5.9–14.6)	13.4 (8.6–18.1)		10.5 (4.6–16.3)	18.5	
ANC in both the Public and Private Sectors, %	1.0 (0.2–1.9)	1.4 (0.4–2.5)		0.7 (0–1.8)	1.4 (0.3–2.4)	
Average wait time at the health facility before being seen by the ANC provider (minutes)	93 (83–103)	85 (77–92)	0.02	92 (81–104)	100 (88–112)	0.06
Total average time at the health facility for ANC (minutes)	141 (129–153)	130 (119–141)	0.01	137 (125–149)	143 (129–157)	0.15
Total time away from home to attend ANC (minutes)	212 (192–233)	197 (182–212)	0.01	210 (194–226)	217 (202–232)	0.17
Total time in care at HF	47.6 (42.7–52.5)	45.6 (40.4–50.8)	0.29	44.7 (38.4–50.9)	43.3 (40.0–46.6)	0.48
Cost (one-way) to get to HF (mean)	360 (265–455)	298 (240–355)	0.07	290 (231–348)	338 (282–395)	0.003
Overall Quality of Care (composite all 7 interventions)	36.8 (31.3–42.3)	38.7 (33.4–44)	0.61	46.9 (40.5–53.3)	39.8 (31.3–48.2)	0.16
Blood pressure measured at least once, %	93.1 (90.8–95.5)	95.3 (93–97.5)	0.18	96.2 (94–98.4)	96.1 (93.8–98.4)	0.92
Urine sample taken at least once, %	92.7 (90.6–94.9)	94.1 (91–97.1)	0.48	93.5 (90.7–96.4)	94.9 (92.9–97)	0.40
Blood sample taken at least once, %	84.1 (80.4–87.8)	88.1 (84.2–92)	0.13	89.7 (86.9–92.4)	91.2 (87.3–95)	0.53
Tetanus protected	64.7 (59.4–70.1)	67.0 (62.6–71.4)	0.51	65.7 (59.8–71.6)	69.5 (65.5–73.6)	0.26
Iron tablets or iron syrup	92.9 (90.1–95.7)	95.2 (93.1–97.3)	0.17	96.1 (94.4–97.8)	97.4 (96–98.9)	0.22
Duration of iron supplementation, weeks	13.6 (12.2–15.1)	15.0 (13.6–16.4)	0.049	16.5 (15.1–17.9)	18.1 (16.8–19.4)	0.01
Treated for intestinal worms, %	78.7 (76.1–81.3)	84.1 (79.9–88.3)	0.03	79.6 (76.4–82.7)	85.8 (81.6–90.1)	0.02
Received an ITN from ANC, %	58.8 (51–66.5)	55.3 (48–62.7)	0.51	69.2 (60.1–78.3)	58.8 (49.2–68.3)	0.09
Location of childbirth						
Public sector	86.9 (81.6–92.2)	82.3 (77–87.7)	0.31	86.7 (80.4–93)	79.6 (74.2–85)	0.06
Private sector	12.2 (7.1–17.4)	16.8 (11.3–22.2)		12.2 (5.7–18.7)	19.8 (14.4–25.2)	
Other	0.9 (0.1–1.6)	0.9 (0.1–1.7)		1.1 (0.2–2)	0.6 (0.1–1.1)	
Mean birth weight, grams	3004 (2925–3084)	3050 (2974–3125)	0.30	2897 (2810–2984)	2927 (2838–3015)	0.51

Nearly all women reported having delivered in a health facility, predominantly in the public sector, with no significant differences by time point or study arm (Table 4). Infant birthweight was not statistically different between women who lived in intervention versus control areas, either at baseline or endline (difference in difference −37.6 g, 95% CI −167 to 92 g) (Table 4).

### Post-partum survey

A total of 2394 women delivering at intervention facilities were approached for inclusion in the survey at the time of delivery; 1550 initiated ANC prior to 25 weeks gestational age, 1542 were between the ages of 15–49 years, and 1506 consented to participate and were included. An average of 84 women were enrolled at each facility, ranging from 27 to 193. Women were an average of 25.8 years old (95% CI 25.4–26.4), had on average 2.9 births (2.7–3.1), 95.2% were married (93.2–97.1), and most (86.2%, 95% CI 79.3–93.1%) had slept under an ITN the preceding night (Table S1).

Among women surveyed post-partum, only one fifth (22.7%; 334) reported having been offered participation in G-ANC. Of those, 55.3% (186) had participated in G-ANC. Women who participated in G-ANC were more likely to have completed secondary education than women who did not participate (Table S1). Of those who did participate in G-ANC, 98.4% reported that they would do so again in a future pregnancy. Women who did not participate in G-ANC gave the following reasons: 29.0% forgot, failed to receive the reminder call from the provider, or had a cancelled group; 21.6% reported that their husband/family objected; 16.9% felt it would be too many visits; 10.8% noted travelling repeatedly to the facility was too costly; 6.8% reported that they did not have time; and 14.9% gave other reasons.

While the overall time spent at the health facility for each ANC visit was longer for women in G-ANC (191 (162–219) minutes versus 175 (151–199) minutes, p = 0.01), the average wait time prior to being seen by a provider was significantly shorter for women in G-ANC (97 (73–122) vs 116 (89–143), p = 0.0003) (Table S2). Overall quality of care (i.e., all seven ANC interventions) was higher among women surveyed in the post-partum survey than in the cross-sectional survey, at 63.0%, with no significant difference between women who attended G-ANC (64.7%, 95% CI 50.0–79.4) and those who attended individual ANC (61.6%, 95% CI 52.2–71.0%; p = 0.62). However, ANC attendance, as well as uptake of IPTp, was significantly higher for women who participated in G-ANC compared to women who did not (ANC4 90.7% (85.9–95.6) versus 57.7% (47.7–67.7; p < 0.001); IPTp3 66.7% (54.2–79.1) versus 45.4 (36.6–54.2); p < 0.001) (Table S2). In addition, birthweight was slightly, but statistically significantly higher, among women who attended G-ANC (3111 gm, 95% CI 3051–3171) as compared to individual ANC (3007 gm, 95% CI 2969–3045; difference 103.9 gm, 42.3–165.5, p-value 0.001) (Table S2).

### Qualitative results

Providers found G-ANC challenging to implement, despite their recognition of the benefits for women. Key challenges to G-ANC implementation were: (1) enrolling enough women to form groups, (2) scheduling G-ANC meetings and identifying a space for the meeting to occur, and (3) implementation challenges, including reminding women to attend each meeting and starting and ending the meetings on time (Table 5). G-ANC implementation was further complicated by the limited number of ANC providers at many of the facilities, which was compounded by the additional workload associated with G-ANC (e.g., forming groups [recruitment, screening, written consent, and enrollment at ANC1], running a number of G-ANC cohorts simultaneously in a health facility, scheduling/re-scheduling and conducting G-ANC meetings, meeting duration). The limited proportion of women in the intervention arm who were offered G-ANC (17.8%, 95% CI 12.8–22.9% in the cross-sectional survey and 22.7%, 95% CI 15.7–29.8%) highlights these challenges.

**Table 5:** Main challenges to G-ANC implementation mentioned by midwives and nurses’ aides in interviews and focus group discussions

Challenge	Description of issues	Illustrative quotes	Potential solutions
Enrolling enough women and forming groups	● Difficulty identifying the requisite number of women who presented sufficiently early in pregnancy ● Difficulty with gestational age estimation to enable group assignment ● Some women moving out of catchment area during pregnancy to stay with mother ● Women’s lack of clarity on the duration of meetings ● Need to have permission from husband to participate ● Cultural barriers around talking about pregnancy	● “There are others who ask, the first question is how long it will take, and they say that when they get home they are going to tell their husbands about it. That they don't know if they will agree.”	● Allow women presenting after 24 weeks to participate in groups ● Keep G-ANC < 24 weeks for first meeting, but add women who come for ANC1 later to Meeting 2 if 24–28 weeks ● Ensure clear communication regarding G-ANC model for women and spouses
Scheduling G-ANC meetings	● Identifying a consistent day and time for a meeting, considering: o on-call schedules for attending deliveries o shortages of providers o staff absences due to off-site trainings ● Effort required to cancel, postpone, or reschedule a meeting ● Ensure women receive reminders to attend	● “As soon as we saw that it can be a little difficult for us, how to take the women and do the G-ANC and at the same time, there will be others who have come for the routine [individual] ANC. We saw that it would be a bit difficult, especially since there are only two of us here.” ● “Sometimes we go through community health workers for mobilization because they are the ones who are in the villages, and they almost know the women of their village.”	● Train all providers in the health centres on G-ANC, especially after staff transfers/changes; use on-the-job mentoring where possible ● Schedule G-ANC on routine days
Implementation challenges	● Shortages of midwives and nurses’ aides in the maternity/delivery units to provide intrapartum care while providers conduct G-ANC meeting ● Space constraints: inadequate size, ventilation ● Having necessary equipment and visual aids available ● G-ANC meetings starting late ● Workload: Number of meetings to be performed, number of G-ANC cohorts in a health centre, duration of the meeting	“…. But when there are deliveries, I have to leave them [the women at G-ANC] first, go and finish the delivery, since I am alone in the maternity ward.” “So, you see it’s in the pavilion for [child] vaccination hut, when it’s busy, it’s a bit difficult, sometimes I have to take them in the waiting area of my office… Space [for G-ANC] is not always found. We use the vaccination pavilion and on the days of vaccination, we stay in the waiting area of the office of the responsible midwife and when it is not raining, we go under the mango tree.” “You see today, there are others who are on time and others who are not on time, if we have at least 4 who are on time, they want us to start at the same time and if you wait for the other 30 min to an hour, it's over, the atmosphere is no longer the same as if they were all on time, they are not happy to waste time, they ask to do for themselves and then to do again for others.”	● Set-up tents at facilities as needed ● Start meetings on time, even if all women have not arrived

Specific barriers to enrolling women included women’s concerns about how much time it would take, questions about the exact differences between G-ANC and individual ANC, and opposition from husbands/partners who did not perceive that the benefit of their wives frequently attending G-ANC outweighed the time and cost. Despite this, most women who were offered G-ANC elected to participate (82.6% in the household survey and 55.3% in the post-partum survey). Providers noted that many women could not be enrolled in G-ANC because they did not meet inclusion criteria (e.g., presented for the first ANC visit too late in pregnancy to be eligible for the study or planned to move out of the facility catchment area to stay with their mothers later in pregnancy). In addition, some women noted that they did not feel comfortable discussing their pregnancies with other women from the community:

*“…If I find out I'm pregnant, I'd have to go to the hospital first to confirm it, and if it's confirmed, I can only tell the person I'm intimate with because with this pregnancy issue, you really have to watch out.”*

Meeting scheduling was complicated by staffing shortages and competing responsibilities of trained providers (e.g., on-call schedule for attending deliveries, providing individual ANC). Additionally, unexpected off-site trainings for the COVID-19 vaccination campaign occasionally led to the need to reschedule meetings. Meeting scheduling was easier in larger health centres where more midwives and nurses’ aides were posted and trained in G-ANC facilitation. Some providers noted space constraints, though the study attempted to address this issue by providing tents to create a space where meetings could be held.

As part of the Benin G-ANC model, providers were provided airtime (mobile phone credit) and expected to call women or their husbands/partners before each G-ANC meeting to remind them to increase retention. This was an ongoing challenge due to provider time constraints and the fact that some women did not own a telephone. To address this challenge, some providers put women into pairs, with each woman in the pair reminding her “sister-friend” about the upcoming G-ANC meeting. A few providers asked community health workers (CHWs) to contact women and remind them of upcoming meetings, and some providers encouraged women to tell their husbands/partners to remind them. Despite the reminders, one of the major challenges to implementation was participants arrived late, which delayed starting meetings.

## Discussion

A number of prior studies have demonstrated increased uptake of interventions and retention in care as a result of G-ANC [7, 8, 12]. To the best of our knowledge, this is the first study to assess whether health facilities in SSA can scale the G-ANC model to a degree that ensures community level impact. There was a small, but statistically significant, increase in the total number of IPTp doses in the intervention communities compared to control (DiD = 0.28, 95% CI 0.07–0.48) and women initiated ANC slightly earlier (DiD = 0.32, 95% CI 0.05–0.59), however, no other significant improvements were seen, including in the number of ANC visits attended or the proportion of women who received IPTp3 or IPTp4. Among the small proportion of women who participated in G-ANC, uptake of both ANC (4 or more visits) and IPTp (3 or more doses) was higher at endline, compared to women in individual ANC. Consistent with prior studies [7], there was not a significant difference in overall quality of care (i.e., receiving all seven ANC interventions), however, women who attended G-ANC were significantly more likely to be fully protected from tetanus, have provided a urine sample, and received an ITN at ANC, highlighting the benefits. Nearly all (98%) women enrolled in G-ANC said they would participate again. However, the overall proportion of women interviewed in the endline cross-sectional survey who participated in G-ANC was very low (12%), limiting the ability to measure intervention impact at the community level.

Facility health care providers reported a number of challenges and considerations in implementing the G-ANC model, some of which have been identified previously [11, 19]. These included challenges with enrolling enough women for cohorts and determining gestational age for enrolment [20], scheduling [21], and staffing constraints [11]. Providers cited late initiation of ANC as a key difficulty in constituting a group of women, but data from the cross-sectional surveys suggest that late attendance at first ANC was not common. Only one-fifth of women interviewed in the post-partum survey (all of whom initiated ANC before 25 weeks gestational age) reported having been offered participation in G-ANC, suggesting that providers’ time constraints or interest/attitude may have played a role in the lower than desired enrolment. A substantial proportion of women who were offered G-ANC participation declined for a variety of reasons, including the number of visits, the time and/or costs to travel to the facility and attend ANC, or the perception that their family members would not approve. Additionally, some women reported feeling uncomfortable discussing their ongoing pregnancies in a group. This social norm (to avoid disclosing pregnancy to anyone besides one’s partner) has been documented in Benin previously [22]. Time, travel distance, and cost of ANC attendance have been well described by others as barriers to routine ANC attendance [22, 23]. In Benin, women are charged a consultation fee for every ANC visit, so frequent G-ANC meetings could have been a financial barrier to enrolment. Reducing the cost of ANC attendance [23, 24] and ensuring that unofficial costs do not contribute to the financial burden could go a long way to increasing ANC attendance in general [25, 26], and would likely increase enrolment in G-ANC.

Implementation outside of the trial context would require less additional provider time at ANC1 and less paperwork (i.e., no need for signed informed consent forms) and might lead to improved acceptability among providers. Eluwa et al*.* described successful implementation of a group model in settings with limited health workers in Nigeria, though each facility only implemented four groups, with a total of 257 women, and they do not describe what proportion of ANC1 clients were included [27]. Routine G-ANC implementation might also improve acceptability among women and their families, as more people became familiar with the approach and did not view it as experimental. There are no published trial reports of G-ANC being introduced and implemented at this scale (i.e., aiming to enroll at least 50% of all ANC1 clients in G-ANC) and over such a long time period (> 18 months).

Providers identified a number of solutions to these implementation challenges, including formalizing the dates/times of meetings, ensuring all providers at each facility were trained in G-ANC, utilizing CHWs to provide reminders, and starting the meetings on time regardless of whether all participants were present. Some of these solutions were implemented by providers during the study period. Future implementation should consider co-designing and adapting the G-ANC model with providers to optimize workload, mitigate enrolment challenges, and maximize the benefits of G-ANC for themselves and clients. In addition, future facility selection for implementation should ensure sufficient staff are available to conduct meetings and attend to all other clients or consider dedicated G-ANC teams responsible for G-ANC at multiple facilities. Complementary activities should sensitize husbands/partners on the importance of frequent, routine ANC visits throughout pregnancy.

### Study limitations

The limited enrolment into G-ANC precluded detection of a population-level impact of the intervention. Provider barriers to offering enrolment to more women speak to the feasibility of wider scale implementation, though it is possible that enrolment would be higher if G-ANC were implemented as a routine intervention by the Ministry of Health, rather than as a study. In addition, facilities did not retain systematic information regarding the number of women offered participation in G-ANC and how many refused, thus it is not possible to know for certain if more women would have enrolled if given the opportunity.

While some outcomes improved among women who participated in group ANC as compared to individual ANC, including retention in four ANC visits and uptake of IPTp3, the ability to assess impact at the community level was limited by poor intervention coverage, with only 14.0% of women in the intervention arm reporting participation due to a number of barriers. Although there was no measurable difference in the composite quality of care score between the intervention and control arms, the improved intervention coverage and high level of satisfaction among women who participated in G-ANC suggest that overcoming obstacles to G-ANC recruitment and implementation to make G-ANC available to those who are interested may be worthwhile to improve maternal and newborn health indicators and outcomes in Benin.

## Supplementary Information

Supplementary material 1.

## Data Availability

All data relevant to the study are included in the article or uploaded as supplementary information. The datasets used and/or analysed during the current study are available from the corresponding author on reasonable request.
