# Supplementary material for: Assessing the community-level impact of group antenatal care on uptake of intermittent preventive treatment for malaria in pregnancy in Atlantique Department, Benin, 2021–2023: a cluster randomized controlled trial
Source: Malar J. 2025 Jul 1;24:205. doi: 10.1186/s12936-025-05412-8 (PMC12211219; doi:10.1186/s12936-025-05412-8)
Supplement: Supplementary file 1 — Supplementary material 1. [file 12936_2025_5412_MOESM1_ESM.docx]

**Supplement to “Assessing the impact of group antenatal care on uptake of intermittent preventive treatment in pregnancy in Atlantique Department, Benin: a cluster randomized controlled trial"**

**Supplemental Methods**

*G-ANC implementation*

G-ANC materials were translated into French and adapted to the Beninese context through a stakeholders’ workshop in January 2021 using Jhpiego’s G-ANC model (Figure S2)[1]. Each intervention facility was provided with G-ANC facilitation materials, automated blood pressure devices and weighing scale for self-assessments, plastic chairs, a privacy screen, exam bed, and some basic supplies. Several intervention HFs also received a tent to create a meeting space. The study provided airtime to providers to call women in G-ANC before each meeting to remind them to attend. Refreshments were provided at the end of every meeting. Group-ANC registers were used to track cohort participants’ attendance, including which participants had been reached with a reminder in advance of each meeting, whether or not they attended, and IPTp administration. While individual pregnant women were consented and enrolled into GANC, the study did not collect or abstract the individual-level data on women in either intervention or control facilities from the ANC registers. Instead, data collection occurred during a household survey among recently delivered women in study facility catchment areas to assess ANC care-seeking practices including MiP prevention interventions and exposure to G-ANC, as described below.

**Training, monitoring, and supervision**. ANC providers at all 40 study sites received a half-day onsite ANC technical update from between December 2020 and March 2021 to standardize basic care, including IPTp and gestational age estimation. Three providers per intervention facility (total 60) were invited to the G-ANC facilitation training. Due to staff shortages in many facilities, only 54 providers (39 nurses and midwives and 15 health aides [MA]; 1-4 providers per facility) serving the 20 intervention facilities participated in the five-day hands-on training on G-ANC facilitation in February 2021, including an orientation to study procedures and scheduling women to join an appropriate cohort. In addition, two health officials per health zone were trained (total of six supervisors). Additional G-ANC training sessions were conducted in December 2021 and January 2022 for 12 newly assigned providers as staff transfers left six intervention facilities without enough providers to conduct G-ANC. After training, providers in the intervention facilities were supported to begin the intervention. Intervention facilities were encouraged to enroll as many women into G-ANC as possible.

Group-ANC specific registers were used to track cohort participants’ attendance and IPTp administration. Study staff abstracted monthly attendance data per cohort per meeting in aggregate. Study staff conducted monthly and quarterly visits to intervention and control facilities, respectively, to monitor progress and support providers. During these visits, G-ANC meetings were observed using a fidelity checklist. The study team together with HZ officials conducted quarterly meetings with G-ANC facilitators from intervention facilities in each health zone to discuss challenges and share best practices. Study staff also maintained a study log to record events that may have affected the study (such as staff transfers, etc.).

**Participant recruitment adjustments.** Enrollment in G-ANC was lower than anticipated, in part due to the COVID-19 pandemic, limited staff availability, and the time available to perform administrative tasks such as obtaining consent from women during ANC clinics, scheduling meetings, and follow up with groups. Thus, in November 2021, five research assistants (RA), all of whom had midwifery training, were hired to support facilities with administrative duties for the study. In August 2022, ten additional RAs were added to assist with a post-partum survey and study activities.

*Baseline and endline cross-sectional surveys and post-partum survey.*

Experienced staff were hired and trained for 4 days to conduct baseline and endline household surveys during November 2-December 12, 2020, and October 24-December 2, 2022, respectively. Prior to survey initiation, staff, all of whom had completed at least some university studies and most of whom had bachelor’s degrees, were trained by the study team in human subjects’ research, the study objectives, standard operating procedures, collection of data using study tools, and obtaining informed consent. To ensure language accuracy, Fon (Fongbe) language tools were translated from the French by an official Beninese French/Fon interpreter. Additionally, training included a detailed review of the questionnaires to ensure that all interviewers understood the questions and asked them in the same way. During household survey training, which included the translator, all data collectors read aloud and practiced administering survey tools in both French and Fon to ensure standardization, and the Fon translator coached the interviewers on how to accurately explain any medical and research concepts that might be unfamiliar.

The CDC’s open-source Sampling app was used to delineate enumeration areas (EA) in catchment areas of the 40 study health facilities, and randomly select 63 households in each of those areas. To assure that at least 33 of the households included a woman aged 15-49 who had experienced a live birth in the previous 12 months, enumerated households were classified by whether there was a resident meeting this criterion.

During the two days of questionnaire administration in each EA, a standardized questionnaire to collect information on demographics, malaria prevention and ANC experiences was administered to consenting women who had experienced a birth in the previous 12 months, or, in households without such a member, to one consenting adult household member. Households in which residents were absent were revisited at least twice before being declared absent; these households were not replaced with another from the list, nor was any replacement made in the case of consent refusal. Malaria RDTs were administered by trained community health workers to all children aged six—59 months after obtaining caregiver consent; those testing positive were treated according to national guidelines. Data was collected on electronic tablets using CommCare software (version 2.52, Dimagi) and uploaded daily to a CommCare database. Survey supervision was provided by study staff.

*Data management and analysis*

Data collection tools for the baseline, endline and delivery surveys were pre-programmed with validation and logic checks using CommCare electronic data collection software. ANC data was collected by providers on paper registers and subsequently entered in a CommCare database by study staff. Data was stored on password-protected laptops and tablets, with restricted access to questionnaires and data by study staff only. Systematic routines were developed to check for data entry discrepancies, range, and consistency. Discrepancies were resolved by reference to the original data collection forms.

Statistical analyses were performed using SAS version 9.4 (Charlotte, NC). A difference in differences analysis was done using proc genmod to estimate the change in the proportion of women receiving both ANC and IPTp from baseline to endline in intervention versus control facilities. The effects of gravidity, ITN use, marriage, education, age, and socioeconomic status were assessed. For the cross-sectional survey analysis, only socioeconomic status was retained in the models as it was the only significant confounder. The post-partum survey was analyzed using proc surveyfreq to account for clustering and weighting.

*Semi-structured interviews and focus group discussions*

The entire qualitative research team consisted of 6 interviewers, 5 women and 1 man, each assigned to administer two types of guides for qualitative data collection. In this article we report on data collected for two guides, one for semi-structured interviews with health care providers and health officials, and one for focus groups with women who attended at least one G-ANC session. The qualitative research had an objectivist epistemology. The methodology was informed by the background of the study team in ethnography, but not fully ethnographic.

These semi-structured interviews and focus groups were conducted by two female qualitative interviewers trained in the social anthropology of health and qualitative research. One of them had a PhD and the other a MA degree, both in sociocultural anthropology. The study staff conducting the interviews had no prior relationship with those being interviewed. The reasons for conducting the research were clearly stated in the consent form which all participants signed prior to participation; no information was given on the researcher’s personal background or reasons for being involved in the study. Research staff were carefully selected by the team based on their CVs and did not have any obvious bias that would have favored a specific outcome of the research. The orientation of the qualitative interviewers consisted of two days of interactive training and classroom practice with application of the interview guides, then one day of pilot testing at the field site, then one day of debriefing on the pilot testing, and revision of the data collection guides.

Three of the seven health officials interviewed were male, the others were female. All health care providers and all focus group participants were female. The focus group participants had similar personal characteristics to the household survey respondents (Table 2). The duration of interviews with health officials and health care providers in general was one hour. In some cases, the interview unfolded over 2 to 3 hours if the health care provider was interrupted and had to step away to address the needs to people arriving for care, or women in labor. The focus groups generally lasted 1.5 hours. Data saturation could not be assessed during data collection, but saturation for the different guides was observed during analysis. Transcripts were not returned to participants for comments or correction. Results were shared with local health officials and health care providers during dissemination meetings, and further feedback was recorded at that time.

Qualitative transcripts were analyzed through a variation on framework analysis. Through initial reading of the transcripts by three members of the study team, inductive codes were created. An analytic questionnaire was created in Google Forms that contained closed-ended questions corresponding to questions in the guides, and also deductive and inductive codes. After data entry in the form, descriptive statistics of the closed-ended questions, and further analysis and characterization of the quotes entered for each code were carried out, followed by discussion of the findings for each code. This article presents both quantitative and qualitative results from the study. Due to word limits, only selected major findings are presented from the qualitative data. Other major findings and minor findings will be communicated in other articles from this study that are being submitted for publication.

**Supplemental References**

1. Kabue MM, Grenier L, Suhowatsky S, Oyetunji J, Ugwa E, Onguti B, Omanga E, Gichangi A, Wambua J, Waka C, et al: **Group versus individual antenatal and first year postpartum care: Study protocol for a multi-country cluster randomized controlled trial in Kenya and Nigeria.** *Gates Open Res* 2018, **2:**56.

**Supplemental Tables**

**Table S1. Demographic characteristics of women in the post-partum survey**

|  | Individual ANC | G-ANC | p-value |
| --- | --- | --- | --- |
| ***Total*** | *1320* | *186* |  |
| **Mean age, years (SD)** | 25.8 (25.3-26.4) | 26.1 (25.1-27.1) | 0.59 |
| **Married/ living together, n (%)** | 94.8 (92.6-97.0) | 97.6 (94.8-100) | 0.18 |
| **Education** |  |  |  |
| No education (1) | 34.0 (28.3-39.8) | 23.7 (15.8-31.7) | 0.004 |
| Primary (2) | 38.7 (33.4-43.9) | 40.0 (31.7-48.3) |  |
| Secondary or higher | 27.3 (21.9-32.7) | 36.3 (26.4-46.1) |  |
| **Slept under an ITN last night** | 85.5 (78.1-93.0) | 91.3 (85.7-96.9) | 0.11 |
| **Parity** |  |  |  |
| 1 pregnancy | 26 (21.3-30.8) | 23.3 (17.8-28.8) | 0.54 |
| 2 pregnancies | 21.5 (18.2-24.8) | 24.7 (20.1-29.3) |  |
| 3 pregnancies | 52.4 (48.9-55.9) | 52 (45.1-58.9) |  |
| **Prior pregnancies, mean (SD)** | 2.9 (2.7-3.1) | 2.9 (2.6-3.2) | 0.72 |

**Table S2. Coverage of ANC and IPTp among women in the post-partum survey, by receipt of G-ANC vs individual ANC**

|  | **Individual ANC** | **G-ANC** | **p-value** |
| --- | --- | --- | --- |
| **Total** | **1320** | **186** |  |
| **ANC2** | 94.7 (91.8-97.6) | 100 (100-100) | - |
| **ANC3** | 80.3 (73.2-87.4) | 97.2 (94.8-99.6) | 0.001 |
| **ANC4** | 57.7 (47.7-67.7) | 90.7 (85.9-95.6) | 0.0003 |
| **ANC6** | 19.9 (12.1-27.6) | 61.6 (46-77.2) | 0.003 |
| **ANC8** | 5.2 (1.5-8.8) | 29.6 (8.5-50.7) | <.0001 |
| **IPTp1** | 97.8 (95.3-100) | 85.9 (74.1-97.7) | 0.10 |
| **IPTP2** | 76.9 (70.4-83.5) | 81.2 (69.6-92.8) | 0.48 |
| **IPTP3** | 45.4 (36.6-54.2) | 66.7 (54.2-79.1) | <.0001 |
| **IPTP4** | 16 (11.3-20.6) | 44.7 (33.2-56.3) | <.0001 |
| **IPTP5** | 3.1 (1.9-4.4) | 20.3 (10.4-30.1) | <.0001 |

*All but one woman in the individual ANC group attended at least 1 ANC visit, and only 5 women (all individual ANC attendees) attended only 2 ANC visits. Due to having no women in the G-ANC group who attended only 1 or 2 ANC visits, no statistics can be calculated for these outcomes.

**Table S3.** Quality of care during pregnancy, post-partum survey

|  | Individual | G-ANC | p-value |
| --- | --- | --- | --- |
| Total | 1320 | 186 |  |
| Proportion of women who attended any ANC* | 99.9 | 100 |  |
| Proportion receiving ANC in the Public Sector only | 97.7 (96.4-99) | 99.1 (97.7-100) | - |
| Proportion receiving ANC in the Private Sector only | 0.9 (0.4-1.4) | 0 |  |
| Proportion receiving ANC in both Public and Private Sectors | 1.4 (0.2-2.6) | 0.9 (0-2.3) |  |
| Average wait time at the health facility before being seen by the ANC provider (minutes) | 116 (89-143) | 97 (73-122) | 0.0003 |
| Average total time at the health facility for ANC (minutes) | 175 (151-199) | 191 (162-219) | 0.01 |
| Average total time away from home to attend ANC (minutes) | 258 (218-297) | 295 (235-355) | 0.0004 |
| Cost (one-way) to get to HF (mean) | 263 (236-291) | 240 (217-263) | 0.04 |
| **Received Quality Care (composite, received all 7 ANC interventions):** | 61.6 (52.2-71.0) | 64.7 (50.0-79.5) | 0.62 |
| Blood pressure measured at least once (proportion) | 99.9 (99.8-100) | 100 (100-100) | - |
| Urine sample taken at least once (proportion) | 95.4 (92-98.8) | 98.8 (97-100) | 0.01 |
| Blood sample taken at least once (proportion) | 90.4 (82.6-98.1) | 91.3 (83.6-99.1) | 0.67 |
| Infant protected from tetanus | 79.3 (72.5-86.0) | 86.0 (78.0-94.1) | 0.03 |
| Iron tablets or iron syrup (yes/ no) | 99.7 (99.4-100) | 100 (100-100) | - |
| Duration of iron supplementation during pregnancy | 26.7 (25.0-28.3) | 24.1 (21.0-27.2) | <.0001 |
| Proportion treated for intestinal worms | 90.3 (86.3-94.2) | 92.8 (87.5-98.2) | 0.26 |
| Proportion who received an ITN from ANC | 79.8 (71.0-88.5) | 88.6 (80.0-97.3) | <.0001 |
| Mean birth weight | 3007 (2969-3045) | 3111 (3051-3171) | 0.001 |

*Only one woman reported not attending ANC

**Figure S1.** Map of Atlantique Dept with intervention and control facilities

**
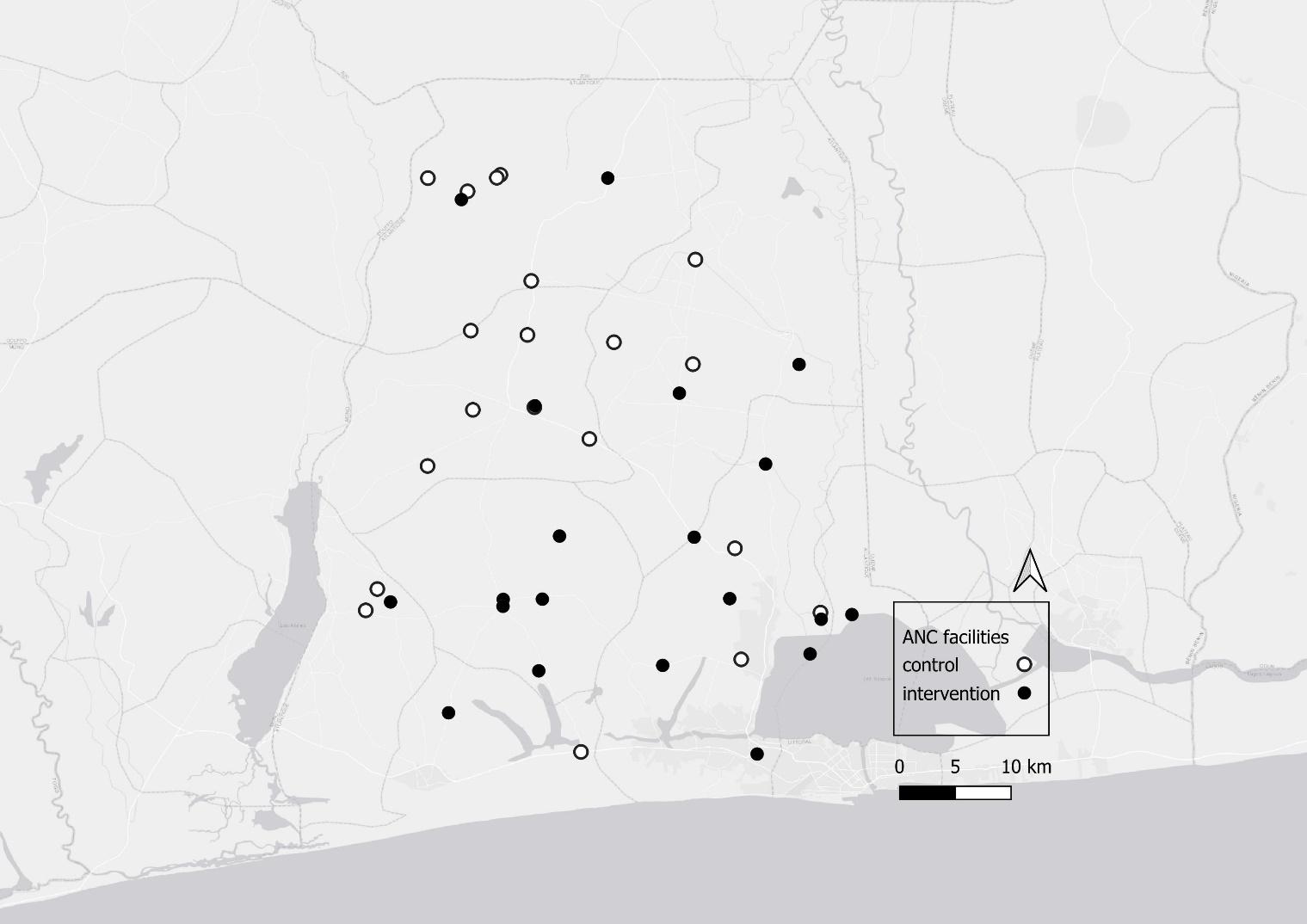
**

**Figure S2. Proposed G-ANC Intervention with Timing and Illustrative Content of Meetings**


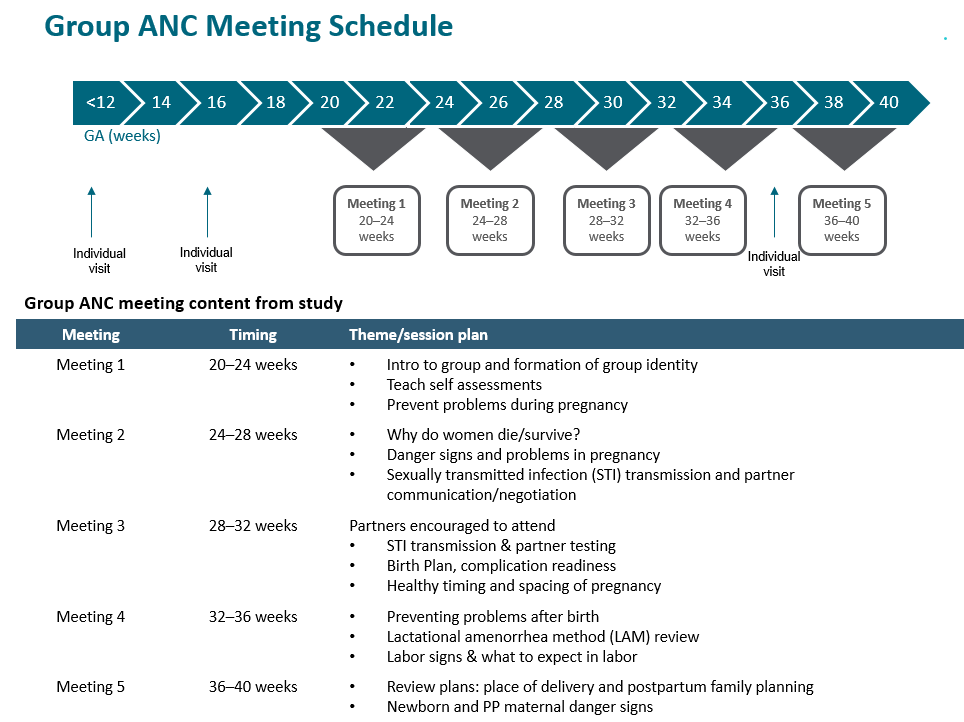


NOTES: National ANC guidelines recommend a minimum of 8 ANC contacts. Per recommendations, women should have multiple ANC contacts in the third trimester at 30, 34, 36, 38, and 40 weeks GA, thus women will be reminded to come for additional individual ANC visits between and after the 5^th^ meeting as needed.

The recommendation for IPTp in Benin is to start at 16 weeks GA and then have monthly doses. Women who came for their first ANC visit at 13–14 weeks GA were encouraged to return for an individual ANC visit at 16 weeks GA, before the first group meeting.
